# Flourishing despite Chronic Obstructive Pulmonary Disease (COPD): Findings from a Nationally Representative Survey of Canadians Aged 50 and Older

**DOI:** 10.3390/ijerph192316337

**Published:** 2022-12-06

**Authors:** Sally Abudiab, Esme Fuller-Thomson

**Affiliations:** 1 Factor-Inwentash Faculty of Social Work, University of Toronto, Toronto, ON M5S 1V4, Canada; 2 Institute for Life Course and Aging, University of Toronto, Toronto, ON M5S 1V4, Canada; 3 Department of Family and Community Medicine, University of Toronto, Toronto, ON M5G 1V7, Canada

**Keywords:** complete mental health, absence of mental illness, COPD

## Abstract

Chronic Obstructive Pulmonary Disease (COPD) is a leading cause of mortality and is often associated with serious disability and depression. Little is known about the characteristics of those who are in complete mental health (CMH) despite having COPD. This study’s objectives are to: (1) estimate the prevalence and odds of absence of psychiatric disorders (APD) and CMH among older adults that reported having COPD, compared to their peers that did not; (2) identify factors associated with APD and with CMH. Bivariate and logistic regression analyses were conducted using the nationally representative Canadian Community Health Survey—Mental Health. The results indicate that there was a significantly (*p* < 0.001) lower prevalence of APD (86.7% vs. 95.0%) and CMH (66.7% vs. 77.0%) among older adults aged 50+ with COPD (*n* = 703) compared to those without COPD (*n* = 10,189). Half of the sample was female (50.5%) and the majority of whom were under age 70 (62.5%). Factors significantly (*p* < 0.05) associated with higher odds of APD and of CMH among older adults with COPD include being married, having a confidant, being physically active, and having no lifetime history of major depressive disorder or generalized anxiety disorder. For every additional adverse childhood experience, the odds of APD declined by 31%. The majority of those with COPD are mentally flourishing despite having this disabling and life-threatening disorder. These findings underline the importance of targeted interventions and outreach to those most vulnerable to poorer mental health outcomes including the socially isolated.

## 1. Introduction

Chronic obstructive pulmonary disease (COPD) refers to a group of progressive lung diseases that are characterized by persistent respiratory symptoms and airflow obstruction [1], whereby affected individuals find it progressively harder to breathe. In the United States, COPD affects 1 in 15 individuals, and is the third leading cause of death with an estimated annual cost of $32 billion in patient-related care costs in 2010 [2]. Globally, the social and economic burden of COPD is projected to increase in coming decades because of the aging of the population [2] and continued exposure to COPD risk factors, such as cigarette smoking, air pollution, and occupational hazards. Thus, COPD presents as a critical public health challenge.

COPD has a high comorbidity with numerous mental health problems, including depression, anxiety, and substance use disorders [3]. COPD patients with co-occurring mental illness often have more severe morbidity and greater premature mortality than COPD patients without mental illness [3]. For example, COPD patients with co-occurring anxiety and depression have an increased risk of symptom exacerbations, self-reported functional limitations, and poorer overall health status [4,5]. This is particularly concerning for those with severe COPD, where the estimated prevalence of comorbid depression is approximately 80% [6]. Additionally, a recent systematic review found that individuals with COPD are approximately twice as likely to commit suicide in comparison to those without COPD [7]. Furthermore, substance use disorders have a higher comorbidity with COPD [8], and have been found to be associated with greater healthcare utilization and associated costs among COPD patients [9].

When examining the relationship between COPD and mental health outcomes, there are several factors that are associated with both COPD and mental illness that may confound the association, including demographics, socioeconomic status, social support, physical activity, coping strategies, adverse childhood experiences, and mental health history. A brief overview of these factors as they relate to both COPD and mental illness is provided below.

### 1.1. Factors Associated with COPD

Those with COPD are more likely to be racialized [10], older [11], have lower education, lower household income, and lower composite SES index [12,13]. Physical activity levels in individuals with COPD are significantly lower than in those without COPD [14,15], particularly among those with severe COPD [16]. Further, individuals with COPD have a higher prevalence of insomnia when compared to individuals without COPD [17].

### 1.2. Factors Associated with Mental Health Problems among Those with COPD

The existing literature demonstrates that there are numerous complex and interrelated factors that may influence both COPD and complete mental health (CMH). Individuals with COPD frequently experience co-occurring symptoms of depression and anxiety [18,19]. Previous studies indicated that anxiety and depressive symptoms in COPD patients were correlated with various factors including age, gender, severity of COPD, general health status, smoking, physical endurance, and social functioning [19,20]. In the general population, those who have a lifetime history of depression or anxiety have been found to be significantly less likely to be in complete mental health (CMH) when compared to those with no lifetime history of either illness [21].

COPD is associated with numerous other physical health conditions, including chronic pain [22], functional limitations [23], and insomnia [24], which are also associated with serious mental illnesses like major depressive disorder [25]. Approximately 20–40% of insomnia cases are related to depression, anxiety, or psychological stress [26]. Importantly, anxiety and depression can precipitate or worsen insomnia, characterizing a bidirectional relationship between mental illness and insomnia [17]. Chronic pain and insomnia are both related to poor-quality sleep which can negatively influence and exacerbate perceptions of pain [27]. Chronic pain is also associated with exaggerated negative cognitive and emotional responses, as well as decreased frequency of physical activity [28]. Individuals with chronic pain and insomnia tend to have worse mental health than those without [29].

Adverse childhood experiences (ACEs), such as abuse, neglect, and exposure to domestic violence in childhood, are important risk factors for the development of lung diseases, such as COPD in adulthood [30], particularly among women [31]. Religiosity and/or spirituality can be considered coping strategies, especially for those with chronic health conditions [32]. Another researcher examined religiosity and spirituality in people with COPD and found that, overall, people with COPD were more likely to employ negative religious coping, or believe that they were being punished by God or abandoned [33].

The presence of social support, via a marriage partner or a close friendship/kinship, is associated with positive physical health outcomes in a wide range of chronic physical health conditions [34,35], as well as a lower prevalence of mental illness, such as depression [36]. In a scoping review, researchers examined the relationship between social support and COPD outcomes and found evidence supporting an association between social support and positive mental health outcomes and self-efficacy among COPD patients [37]. However, there was inconsistent evidence on the association between perceived social support and quality of life, physical functioning, and self-rated health [37].

Cigarette smoking is the leading risk factor for COPD [38]. Smoking also has a high comorbidity with mental illness, particularly depression and anxiety [39,40,41].

These factors have been included in the current analyses in order to minimize confounding bias and allow the analysis to more accurately illuminate the independent association between COPD and CMH.

### 1.3. Complete Mental Health (CMH)

Research on mental health outcomes among COPD patients has been largely deficit-focused, emphasizing negative outcomes among this population [5,42]. Recently, there has been a shift in some mental health research toward a more holistic approach to mental well-being and flourishing (e.g., [43]). This perspective can be conceptualized through Keyes’ [44] concept of CMH. The CMH approach emphasizes that people who are free from mental illness are not necessarily in a state of positive mental health [45]. The CMH model that is utilized in the current study is comprised of three components: (1) the absence of psychiatric disorders (APD) in the preceding year (i.e., depressive disorders, anxiety disorders, bipolar disorders, substance dependence, suicidal ideation); (2) the presence of happiness or life satisfaction daily or almost daily in the preceding month; and (3) the presence of social and/or psychological well-being daily or almost daily in the preceding month. Examining CMH allows clinicians to consider the absence of psychopathology, in addition to the presence of emotional, psychological, and social well-being [44]. APD and CMH are both used in this paper to advance our understanding of mental well-being in older adults with COPD.

### 1.4. Study Objectives

The objectives of this study are the following:

(a) To estimate the prevalence and odds of absence of psychiatric disorders (APD) and of CMH among adults 50 years and older who reported having COPD, compared to their peers who did not;
(b) To identify factors associated with APD among older adults who reported having COPD; and
(c) To identify factors associated with CMH among adults who reported having COPD.

We hypothesize that those with COPD will have lower prevalence and odds of APD and CMH than their peers without COPD. We also hypothesize, based on the literature discussed above, that among those with COPD the factors associated with positive mental health will be being married, having a confidant, being physically active, and having no lifetime history of major depressive disorder or generalized anxiety disorder.

## 2. Materials and Methods

As has been reported elsewhere [43,46], the current study was undertaken using nationally representative data from the 2012 Canadian Community Health Survey—Mental Health (CCHS-MH). The target population of the CCHS-MH includes 97% of the population who are over the age of 15 and reside in the 10 Canadian provinces. The response rate was 68.9% nationally [47].

### 2.1. Sample

A sub-sample of the full CCHS-MS dataset was restricted to those aged 50 and older (*n* = 10,892 respondents) who had complete data on all the variables used in the fully adjusted model. Of these, 703 respondents reported they had been diagnosed by a health professional with “chronic bronchitis, emphysema or chronic obstructive pulmonary disease or COPD”. These respondents with COPD comprised the subsample for the logistic regression analysis of those with COPD.

A total of 1621 respondents were excluded from the analysis because of missing data, which decreased the sample size by 13%. In order to permit the reader to compare the characteristics of those with and without missing data, chi-square analyses were conducted.

Respondents with missing data did not differ significantly from respondents with complete data with respect to the prevalence of COPD (5.5% vs. 4.9%; *p* = 0.30), to having insomnia (18.8% vs. 17.0%; *p* = 0.09), to have ever experienced depression (10.9% vs. 10.0%; *p* = 0.3) or anxiety (10.0% vs. 8.7%; *p* = 0.1).

Respondents with missing data, when compared to respondents without missing data, had a lower prevalence of APD (88.2% vs. 94.6%; *p* < 0.001), CMH (72.7% vs. 76.5%; *p* = 0.03), and were less likely to have received a post secondary degree (51.7% vs. 58%, *p* < 0.001), and to be married (63.7% vs. 72.4%, *p* < 0.001).

Those with missing data were more likely than those with complete data to be female (58.6% vs. 50.5%; *p* < 0.001), be racialized (17.1% vs. 13.9%; *p* < 0.05), to be without a confidant (6.9% vs. 3.0%; *p* < 0.001), to have never smoked (37.1% vs. 30.7%; *p* < 0.001), to not do any moderate/vigorous physical activity (40.1% vs. 31.3%; *p* < 0.001), to have pain that prevents at least some of their activities (25.5% vs. 19.6%; *p* < 0.001), to believe that spiritual values play an important role in ones life (74.8% vs. 71.6%; *p* < 0.05), and to have never misused drugs or alcohol (84.2% vs. 79.4%; *p* < 0.001).

### 2.2. Measures

**Outcome Variables:**

Two different outcome measures were evaluated; (a) *APD in the past year* was based on no suicidal ideation in the past year nor depressive episode, anxiety disorders, bipolar disorders, nor alcohol or drug dependence including cannabis and other drugs. World Health Organization’s version of the Composite International Advances in Preventive Medicine Diagnostic Interview (WHO-CIDI), a structured diagnostic interview that generates past-year diagnosis according to the Diagnostic and Statistical Manual of Mental Disorders, Fourth Edition (DSM-IV) and the international Classification of Disease (ICD-10) was used to derive these variables. Finally, (b) *CMH*, measured as a binary variable, consisted of three parts: (1) the absence of psychiatric disorders in the past year, as was described above; (2) emotional wellbeing (i.e., life satisfaction or happiness), and (3) social and psychological well-being. The latter two were assessed using the Mental Health Continuum-Short Form (MHC-SF) [47]. The MHC-SF examines positive mental health using 14-items that assesses psychological well-being (e.g., during the past month, how often did you feel that you liked most parts of your personality?), emotional well-being (e.g., during the past month, how often did you feel: happy and/or satisfied with your own life?), and social well-being (e.g., during the past month, how often did you feel that you had something important to contribute to society?) [47,48]. The MHC-SF has well established psychometric properties [48]. Respondents were categorized as being in CMH if they stated at least 1 of the 2 measures of emotional well-being (i.e., happiness and/or life satisfaction in past year) and a minimum of 6 of the 11 measures of psychological and/or social wellbeing “every day” or “almost every day” during the past month in tandem with the absence of any of the above listed forms of mental illness in the past year. Additional information can be found at Statistics Canada [47]. Our version was slightly modified by removal of one-question from the original 14-item instrument. The original version had included the question whether the respondent was “interested in life” in addition to “happy” and “satisfied with life” in the “emotional well-being” category. We felt that it was possible to be interested in life without being in optimal mental health and therefore removed it from the measure which resulted in the instrument having only 13 items now. Furthermore, in a previous study of the psychometric properties of the MHC-SF, “interest in life” had markedly lower factor loading on emotional well-being than either “happy” or “satisfaction with life” [48]. The internal consistency (Cronbach’s alpha) for the 13-items was high (0.89).

**Predictor Variables:**

*Demographic variables* included sex (male versus female), age (in decades), education and self-reported race (Non-Aboriginal white versus racialized and/or Aboriginal). Due to confidentiality issues, the public-use data set of the CCHS-MH does not release information on specific race/ethnicities.

*Socioeconomic status* was calculated based on the highest level of education completed (less than post secondary degree, post secondary degree) and household income. In line with Statistics Canada’s measure of household income, this study used a ratio associated with the national low-income cut-off, which takes into account the quantity of people who reside in a home and the size of the neighborhood (lowest 10% 11–50%; top 50% of household income).

*Social support* measures included marital status and having an advisor/confidant available for critical life decisions and situations. Marital status was measured according to the following two categories: Married/common-law versus divorced/widowed/never married. Having a confidant available for critical life decisions and experiences was ascertained using those who agreed or strongly agreed to the statement “There is someone I could talk to about important decisions in my life”.

*Physical health measures* included using a derived variable from Canada Statistics [47], smoking status was dichotomized into never smoked, ever smoked. Obesity was categorized into two groups: a body mass index (BMI) of 30 or higher (obese) versus less (not obese). The BMI was calculated from self-reported weight and height. Further details about these measures are available from Statistics Canada [47].

Insomnia was based upon the question “How often do you have trouble going to sleep or staying asleep?” (most or all of the time vs. none/a little/some of the time). Debilitating chronic pain was based on two questions: (1) “Are you usually free of pain or discomfort” (no/yes); and (2) “How many activities does your pain or discomfort prevent?” (none, a few, some, most). Those who reported no to question 1 and ‘most’ to question 2 were defined as having debilitating chronic pain.

Two *coping strategies* were examined: (1) Religious coping was assessed based on the question ‘To what extent do your religious or spiritual beliefs give you the strength to face everyday difficulties?’ (a lot/somewhat/a little versus not at all). (2) Physical activity level was based on the number of times participants reported spending doing vigorous or moderate physical activity within the past 7 days. For more information on this variable please refer to Statistics Canada [47].

Two *adverse childhood experiences* were summed: Childhood Sexual Abuse (CSA): Childhood sexual abuse was measured by the questions “How many times did an adult force you or attempt to force you into any unwanted sexual activity, by threatening you, holding you down or hurting you in some way?” (never vs. ever). Childhood Physical Abuse (CPA) was assessed by asking respondents if an “adult had slapped them on the face, head or ears, or hit or spanked them with something hard to hurt them at least three times and/or pushed, grabbed, shoved or threw something at them to hurt them at least three times and/or an adult had at least once kicked, bit, punch, choked, burned, or physically attacked them”.

*Lifetime mental health history* include anxiety, depression and substance dependence. Individuals were categorized as having an anxiety disorder if they met the WHO-CIDI criteria for Generalized Anxiety Disorder at some point in their lives. Respondents who met the criteria for lifetime Generalized Anxiety Disorder reported “(1) excessive anxiety and worry and anxiety about at least two different events or activities that lasted at least six months; (2) finding it difficult to control the worry; (3) the anxiety and the worry were associated with three or more of the symptoms associated with anxiety; (4) the focus of the anxiety and worry was not confined to features of an Axis 1 disorder; and (5) the anxiety, worry, or physical symptoms caused clinically significant distress or significant impairment in social, occupational, or other important areas of functioning” [47].

Respondents were categorized as having a depressive disorder during their lifetime if they met the WHO-CIDI lifetime criteria for Major Depressive Episode. “Respondents who meet the criteria reported: (1) two weeks or longer of depressed mood or loss of interest or pleasure and at least five symptoms associated with depression which represent a change in functioning; (2) that symptoms cause clinically significant distress or impairment in social, occupational or other important areas of functioning; and (3) that symptoms are not better accounted for by bereavement or symptoms last more than two months or the symptoms are characterized by a marked functional impairment, preoccupation with worthlessness, suicidal ideation, or psychomotor retardation” [47]. The WHO-CIDI has strong psychometric properties and excellent validity and reliability. 

The lifetime substance dependence variable was based on detailed measures of lifetime alcohol and/or drug dependence based upon WHO-CIDI scales. These scales have an excellent concordance with clinical interviews (0.83) [49] and have a test-rest reliability above 90% [50].To be defined as substance dependent the respondent must report a minimum of three of the following symptoms: tolerance, withdrawal, increased consumption, attempts to quit, time lost, reduced activities, continued drinking and “(2) a maladaptive pattern of alcohol use as manifested by three (or more) symptoms occurring at any time in the same 12-month period” [47].

## 3. Analysis

In the sample including respondents with and without COPD, bivariate analyses were conducted using chi-square tests for categorical variable (e.g., sex, ethnicity) and independent t-tests for continuous variables (e.g., adverse childhood experiences) by COPD status. The variables included in the analysis were decided a priori based on our extensive review of the literature. The magnitude of the chi-square test is an indication of the effect size, with a Cramer’s V of 0.10 being small, 0.30 is medium, and 0.50 is large [51]. Due to the multiple comparisons conducted in Table 1, to minimize the probability of type 1 error, we divided the 0.05 level of significance by 17, due to the 17 variables analyzed. This results in a cutoff of probability of 0.003 in the chi-square analyses.

Two logistic regression analyses were conducted in the sample including those with and those without COPD, one with APD as the outcome and one with CMH as the outcome, to determine the crude odds of each outcome for those without COPD compared to those with COPD.

In the sample including only respondents aged 50 and older with COPD, two logistic regression analyses were conducted, one with APD as the outcome and one with CMH as the outcome, to determine which factors were associated with each outcome. All the variables described in the measures section were included in each logistic regression analysis.

Data were weighted to account for the probability of selection and nonresponse; however, the sample sizes are provided in their unweighted form.

### 3.1. Results

Table 1 presents the description of those with and without COPD in a population-based sample of Canadians aged 50 and older (*n* = 10,892). Older adults with COPD had a significantly lower prevalence of APD than older adults without COPD (86.7% vs. 95.0%; *p* < 0.001). Older adults with COPD also had a significantly lower prevalence of CMH than older adults without COPD (66.7% vs. 77.0%; *p* < 0.001). Respondents with COPD were more likely to be female and white. Respondents with COPD were also significantly older than those without COPD. Respondents with COPD had a lower household income, were more likely to have ever smoked, to be obese, to have insomnia, to have debilitating chronic pain, and to have a lifetime history of major depressive disorder, generalized anxiety disorder, and a substance use disorder. Respondents with COPD were less likely to have a post-secondary education, to be married, to engage in regular physical activity, and to have someone to confide in. Approximately 41% of the individuals with COPD responded that they had chronic disabling pain compared to 19% of those without COPD. A *t*-test was conducted (not shown in Table 1) on mean number of ACEs by COPD status. Those with COPD had a significantly (*p* < 0.001) higher mean number of ACES than those without COPD (Mean = 0.52; SD = 0.82 vs. Mean = 0.36; SD = 0.62, respectively). All *p*-values in Table 1 were less than the adjusted cutoff of probability (0.003) and therefore reached statistical significance.

The crude (unadjusted) odds of APD for those without COPD was approximately three times higher compared to those with COPD (OR = 2.90, 95% CI = 2.23, 3.78). The crude odds of CMH for those without COPD was 67% higher compared to those with COPD (OR = 1.67, 95% CI = 1.39, 2.01) (Analyses not shown in tables).

Table 2 provides the results of two logistic regression models examining the odds of APD and CMH, respectively, among older adults with COPD (*n* = 703). The odds of APD were approximately doubled for married or common-law respondents compared to single, divorced, or widowed respondents (OR = 2.14, 95% CI = 1.11, 4.15). For those with a confidant, the odds of APD were approximately 8-fold compared to those without a confidant (OR = 7.97, 95% CI = 3.36, 18.93). Respondents who engaged in moderate or vigorous physical activity had quadruple the odds of APD in comparison to their less active peers (OR = 3.95, 95% CI = 1.94, 8.08). Among those with COPD, for every additional ACE, the odds of APD declined by 31% (OR = 0.69, 95% CI = 0.48, 0.98). Individuals who had no lifetime history of major depressive disorder had 11 times higher odds of APD compared to those with a lifetime history of major depressive disorder (OR = 11.27, 95% CI = 5.22, 24.34). Individuals who had no lifetime history of generalized anxiety disorder had 10 times higher odds of APD compared to those with a lifetime history of generalized anxiety disorder (OR = 9.93, 95% CI = 5.03, 19.62). Individuals without a lifetime history of a substance use disorder had quadruple the odds of APD in comparison to those with a lifetime history of a substance use disorder (OR = 4.36, 95% CI = 2.11, 9.05).

As shown in Table 2, columns 4 and 5, among older adults with COPD, white respondents had more than double the odds of CMH comparison to their visible minority peers (OR = 2.76, 95% CI = 1.44, 5.30). Married respondents or those living common-law had 64% higher odds of CMH in comparison to single, divorced, or widowed peers (OR = 1.64, 95% CI = 1.11, 2.42). For those with a confidant, the odds of CMH were approximately seven-fold in comparison to those without a confidant (OR = 6.93, 95% = 3.32, 14.47). Respondents who engaged in moderate or vigorous physical activity had double the odds of CMH in comparison to their inactive or low active peers (OR = 2.16, 95% CI = 1.47, 3.17). Those who had never experienced major depressive disorder in their lifetime had approximately 87% higher odds of CMH than those with a history of major depressive disorder (OR = 1.87, 95% CI = 1.07, 3.26). Those who had never experienced generalized anxiety disorder during their lifetime had triple the odds of CMH than those with a history of generalized anxiety disorder (OR = 3.16, 95% CI = 1.92, 5.20).

Supplemental Tables S1 and S2 provide additional information on unstandardized coefficient (B), standard error of the mean (S.E.), wald, and degrees of freedom (df) for APD and CMH, respectively.

### 3.2. Discussion

The first objective of this study was to examine the prevalence and odds of APD and of CMH among older adults without COPD in comparison to their peers with COPD. In keeping with our hypothesis, the findings of this nationally representative Canadian study indicate that older adults without COPD have much better mental health than their peers with COPD, as evident by their significantly higher prevalence and crude odds of APD and CMH.

There were several factors that separated older adults with and without COPD in the current study. For example, approximately two in five respondents with COPD reported experiencing chronic, disabling pain that impedes their daily activities. This was significantly higher than the approximate one in five respondents without COPD who experienced chronic pain.

The second and third objectives of this study were to investigate factors associated with APD and CMH among older adults with COPD. There were several factors that were found to increase the odds of APD and CMH among this population, in keeping with our hypotheses. The two measures of social support used in this study, marital status and presence of a confidant, were positively associated with APD and CMH. Close social relationships are a key contributor to physical and mental well-being [52]. The stable availability of a close confidant is particularly important for supporting an individual’s emotional well-being, and has been linked to better overall mental health and quality of life [53]. Previous research has found higher levels of depression among older adults who lack close social relationships [54]. Older adults who lack a close confidant or marital partner may also experience higher levels of loneliness [55], which is an identified risk factor for depression and suicidality [56], as well as declines in overall self-rated health [57]. Having social support via a partner or confidant may be particularly important for older adults with serious chronic conditions such as COPD. For example, following a cardiovascular event, older adults who lack a close relationship may have poorer compliance with medical treatment [58], which may result in increased levels of depression and anxiety [59].

Regular physical activity was associated with APD and CMH, aligning with other research on the importance of physical activity to improve well-being among people with COPD [60]. Regular exercise is thought to promote positive mental health outcomes through several mechanisms, including lowering stress hormones like cortisol [61], distracting individuals from negative thoughts [62], and promoting self-efficacy, resilience, and confidence [63].

In this study, there were several factors that were associated with lower odds of APD and CMH, such as having a lifetime history of ACEs, major depressive disorder, generalized anxiety disorder, and substance use disorders.

ACEs are strongly associated with both COPD and CMH. Experiencing ACEs may increase the risk of COPD through maladaptive stress mechanisms and pathways involved in the reduction and premature decline of lung growth and lung function [64]. The association persists even after controlling for such known COPD risk factors, including cigarette smoking, diabetes, and obesity, highlighting the role that ACEs may play in COPD risk [64]. A history of ACEs also have a negative impact on mental health outcomes and have been previously identified as a barrier to being in CMH [65]. The effects of ACEs include higher prevalence of anxiety, violent behaviour, post-traumatic stress disorder, and substance use disorders in adulthood [66,67]. ACEs may impede the development of positive coping strategies and emotional regulation, and exacerbate maladaptive attachment styles in adulthood, ultimately compromising mental health [68].

The finding that lifetime history of major depressive disorder and generalized anxiety disorder were associated with lower odds of APD and CMH among those with COPD aligns with other research on the mental health of COPD patients. A recent study by Yohannes et al. [69] found that certain symptoms of COPD, such as cough and COPD-related weakness were linked to core symptoms of depression (e.g., sadness, lack of pleasure), whereas chest tightness was linked to acute anxiety symptoms (e.g., fear). Depression and anxiety are difficult to diagnose and treat in COPD patients because their symptoms frequently coincide with COPD symptoms [69]. COPD patients with co-occurring depression have been found to have higher levels of anxiety, less social support, higher social stress and more subjective impairment in quality of life than COPD patients without depression [70]. These findings emphasize the need to address individual’s history of mental illness to support the health of COPD patients.

Our findings also indicated that individuals with a lifetime history of a substance use disorder were less likely to be in CMH. Substance use disorders, particularly alcohol use disorders, have been found to be more prevalent among those with COPD when compared to the general population [8].

## 4. Practice Implications

Although the majority of older adults with COPD are flourishing, this study’s findings underline that they are still at a disadvantage compared to their peers without COPD > Given the substantial impact of COPD on the mental health of some older adults, it is important to identify modifiable factors that may be associated with better mental health outcomes among those with COPD, so that interventions can be implemented accordingly. For example, it may be helpful for clinicians to foster social support networks for isolated individuals with COPD, given that that those with a confidant are more likely to be in APD and CMH. This supports other research on the importance of fostering social support to support the health of older adults [71].

Clinicians should also consider ways to foster physical activity among their COPD patients, given the robust evidence of a link between physical activity and improved mental health. Although older adults with COPD may have varying levels of physical ability, it is important for clinicians to encourage COPD patients to engage in some form of regular exercise according to their ability and comfort level. Physical activity training can help COPD patients with comorbid physical and mental health conditions including dyspnea, anxiety, and depression symptoms, as well as enhance their quality of life [72]. However, physical activity is low among the majority of COPD patients [73,74]. Critically, this has been linked to higher healthcare utilization, a worse reported quality of life and premature death. Promising techniques such as goal setting, counselling, daily diary usage, and messaging using smartphone applications, have shown short-term benefit in boosting daily physical activity among COPD patients [75].

The current study also indicates that visible minority respondents were less likely to be in CMH than their white peers, indicating that clinicians may need to provide more accessible mental health support for their racialized patients with COPD. Furthermore, in the therapeutic management of older adults with COPD, proper pain detection and treatment is critical, and pain evaluation should be integrated into routine symptom assessments. Pain is often underrecognized and undertreated in racialized populations, resulting in subpar pain management for this population [76]. When considering that COPD patients often experience chronic pain [28], combined with the high comorbidity between chronic pain and mental illness [77], it is evident that there is a need to address this pain disparity to support the well-being of racialized older adults with COPD.

Health care providers may wish to consider referrals for mental health interventions such as cognitive-behavioural therapy (CBT) for older adults with COPD who are struggling with their mental health. CBT has demonstrated effectiveness for reducing symptoms of mental illness in COPD patients with comorbid depression and anxiety [78,79]. Further research should identify how health care providers can tailor screening and interventions to help COPD patients with comorbid mental illness to support their well-being and improve long-term health outcomes. Given the link between maladaptive physical and social behaviours (e.g., insomnia, ACEs, depression, anxiety) and maladaptive stress responses, strategies to treat this patient population with a trauma-informed strategy is critical. Thus, mental health experts should be integrated into primary care settings to better support individuals with COPD who have experienced trauma. Further, when considering the high prevalence of substance use disorders among COPD patients, strategies to promote tobacco and alcohol reduction and/or cessation should also be made more accessible to COPD patients. To overcome barriers at the patient, health provider, and health system levels, and improve health outcomes, it is important to educate people living with COPD on evidence-based treatments, as well as integrate mental health care practitioners into multidisciplinary outpatient services to maximize health and social outcomes.

## 5. Limitations

The findings of the current study should be considered in light of some notable limitations. First, in this study COPD was based on a self-report of a medical diagnosis. However, many people with COPD have not been diagnosed [80] and some people diagnosed with COPD do not meet the spirometrically validated measure for COPD [81]. Future research would benefit from chart reviews or the use of spirometry for validation. In all likelihood, there is an issue with under-reporting because the prevalence of self-reported COPD found in this study is much lower than that reported in a national study using Canadian Chronic Disease Surveillance System, which indicated a prevalence of diagnosed COPD of 10% among those aged 55–64 rising to 26% among those aged 85 and older [82].

Second, other factors such as physical activity level was based upon self-report, which may have resulted in individuals inaccurately reporting their level of physical activity. Future research needs to use objective measures of physical activity such as a wearable activity tracker (e.g., Fitbit).

Third, the cross-sectional study design prevents the determination of temporality—it is unclear if COPD diagnosis preceded APD and CMH. Fourth, the dataset utilized in the current study excludes older adults who are hospitalized or in long-term care facilities, limiting the generalizability of these findings to community-dwelling older adults who are less severely ill with COPD and presumably less likely to be as impacted by mental health challenges. Such an error would bias the findings towards the null.

Fourth, approximately 13% of those aged 50 and older in the CCHS-MH data set were excluded from the analysis due to missing data on one or more of the variables used in the analysis. Those excluded has significantly lower prevalence of APD and CMH, suggesting that this study may be biased towards positive health outcomes. However, these differences were relatively modest in size, suggesting the bias is unlikely to totally account for the observed results. Importantly, COPD status was not associated with whether or not the respondent was missing from the analysis.

Finally, this study does not include information on whether or not respondents were receiving treatment for a mental health issue, which may influence APD and CMH.

## 6. Conclusions

As discussed in the limitations section above, future research would benefit from a more rigorously designed study with objective measures of relevant characteristics (e.g., physical activity level using activity trackers) and of mental health outcomes using chart review. Despite these limitations, the current study makes a substantial contribution to the literature on the mental health of COPD patients using a large nationally representative sample of Canadian older adults. The results indicate that more than four in every 5 (87%) COPD patients were free of all psychiatric disorders and serious suicidal thoughts and addictions in the preceding year and two thirds were flourishing and in complete mental health. These findings are encouraging for older adults with COPD and their families and the health care professionals caring for them. In addition, this study identifies several factors that are associated with positive mental health outcomes among older adults that reported having COPD, such as physical activity and social support, highlighting critical potential points of intervention to further support the well-being of this population. Furthermore, this study also draws attention to the minority of older adults with COPD who may be more vulnerable to worse mental health outcomes, such as racialized older adults, emphasizing the need for targeted and accessible mental health support. The findings of this study can help guide intervention to support the mental health of this population and learn from those with COPD who are flourishing.

## Figures and Tables

**Table 1:** Description of those with Chronic Obstructive Pulmonary Disease (COPD) and those without COPD in a Population-based Sample of Canadians aged 50 and older (*n* = 10,892) Source: Canadian Community Health Survey-Mental Health.

	Unweighted Sample Size (*n*)	Weighted % of Total	Total % without COPD	Total % with COPD	*p*-Value (Cramer’s V)
**Mental Health Status**					
Absence of Psychiatric Disorders (APD)					
No	744	5.4	5.0	13.3	<0.001 (0.08)
Yes	10,148	94.6	95.0	86.7	
Complete Mental Health (CMH)					
No	2674	23.5	23.0	33.3	<0.001 (0.05)
Yes	8218	76.5	77.0	66.7	
**Demographics**					
Sex					
Male	4796	49.5	49.8	44.1	0.011 (0.02)
Female	6096	50.5	50.2	55.9	
Ethnicity					
Visible Minority	993	13.9	14.2	7.7	<0.001 (0.04)
White	9899	86.1	85.8	92.3	
Age in Decades					
50s	3725	44.4	45.3	26.5	<0.001 (0.09)
60s	3650	30.6	30.4	35.0	
70s	2242	17.2	16.7	25.4	
80s	1275	7.8	7.6	13.1	
**Socioeconomic Status**					
Education					
No post-secondary degree	4923	42.0	41.4	53.4	<0.001 (0.05)
Has post-secondary degree	5969	58.0	58.6	46.6	
Household Income					
Lowest 10% of household income	923	7.9	7.6	13.9	<0.001 (0.08)
11–50%	3984	31.1	30.6	41.9	
Top 50% of household income	5985	60.9	61.8	44.2	
**Social Support**					
Marital Status					
Single/Divorced/Widowed	4693	27.6	26.9	41.4	<0.001 (0.07)
Married/Common-Law	6199	72.4	73.1	58.6	
Presence of a Confidant					
No	375	3.0	2.8	7.9	<0.001 (0.06)
Yes	10,517	97.0	97.2	92.1	
**Physical Health**					
Smoking					
Never	3176	30.7	31.6	13.7	<0.001 (0.08)
Ever Smoker	7716	69.3	68.4	86.3	
Obesity					
BMI < 30	8289	77.4	77.7	71.2	<0.001 (0.03)
BMI ≥ 30	2603	22.6	22.3	28.8	
Sleep Problems					
Never to some sleep problems	8784	83.0	83.6	71.4	<0.001 (0.07)
Most or all of the time sleep problems	2108	17.0	16.4	28.6	
Debilitating Pain					
No pain or no activity prevented by pain	8502	80.4	81.5	59.4	<0.001 (0.10)
Pain prevents few/some/most activities	2390	19.6	18.5	40.6	
**Coping Strategies**					
Religion or Spirituality Used to Cope					
No	2870	28.4	28.5	25.7	0.153 (0.01)
Yes	8022	71.6	71.5	74.3	
Physical Activity					
Moderate/High	7210	68.7	69.8	47.9	<0.001 (0.10)
Inactive/Low	3682	31.3	30.2	52.1	
**Mental Health History**					
Lifetime Major Depressive Disorder					
No	9682	90.0	90.2	86.0	0.002 (0.03)
Yes	1210	10.0	9.8	14.0	
Lifetime General Anxiety Disorder					
No	9835	91.3	91.8	82.4	<0.001 (0.07)
Yes	1057	8.7	8.2	17.6	
Lifetime Substance Use Disorder					
No	8594	79.4	79.9	70.8	<0.001 (0.05)
Yes	2298	20.6	20.1	29.2	

**Table 2:** Odds ratio and 95% Confidence Intervals of Absence of Psychiatric Disorders (APD) and Complete Mental Health (CMH) in Past Year for Those with a History of COPD in a Population-based Sample of Canadians aged 50 and Older (*n* = 703). Source: Canadian Community Health Survey-Mental Health.

	Odds Ratio for (APD) “No Past-Year Mental Illness, Substance Dependence or Suicidality”	95% CI	Odds Ratio of Complete Mental Health	95% CI
**Demographics**				
Gender				
Male (ref.)	1.00	REF	1.00	REF
Female	1.61	(0.71, 3.62)	1.14	(0.74, 1.76)
Ethnicity				
Visible Minority (ref.)	1.00	REF	1.00	REF
White	1.51	(0.45, 5.07)	2.76 **	(1.44, 5.30)
Age in Decades	1.14	(0.80, 1.62)	1.18	(0.96, 1.44)
**Socioeconomic Status**				
Education				
No post-secondary degree (ref.)	1.00	REF	1.00	REF
Has post-secondary degree	0.82	(0.44, 1.55)	1.18	(0.81, 1.71)
Household Income				
Lowest 10% of household income (ref.)	1.00	REF	1.00	REF
11–50%	1.15	(0.46, 2.86)	0.90	(0.51, 1.59)
Top 50% of household income	1.66	(0.64, 4.28)	1.22	(0.68, 2.17)
**Social Support**				
Marital Status				
Single/Divorced/widowed (ref.)	1.00	REF	1.00	REF
Married/Common in Law	2.14 *	(1.11, 4.15)	1.64 *	(1.11, 2.42)
Presence of a Confidant				
Strongly Disagree/Disagree (ref.)	1.00	REF	1.00	REF
Strongly Agree/Agree	7.97 ***	(3.36, 18.93)	6.93 ***	(3.32, 14.47)
**Physical Health**				
Smoking				
Ever Smoker (ref.)	1.00	REF	1.00	REF
Never	0.59	(0.22, 1.59)	1.21	(0.68, 2.15)
BMI (self-reported)				
No (ref.)	1.00	REF	1.00	REF
Yes (Obese)	0.63	(0.31, 1.25)	1.24	(0.81, 1.88)
Sleep Problems				
Most or all the time sleep problems (ref.)	1.00	REF	1.00	REF
Never to some sleep problems	1.38	(0.70, 2.72)	1.23	(0.81, 1.87)
Pain preventing activities				
Pain prevents few/some/most activities (ref.)	1.00	REF	1.00	REF
No pain or no activity prevented by pain	1.18	(0.58, 2.41)	1.44+	(0.97, 2.12)
**Coping Strategies**				
Spiritual Values				
Not very/not at all important (ref.)	1.00	REF	1.00	REF
Very/somewhat important	0.52+	(0.24, 1.12)	1.50+	(0.98, 2.29)
Moderate or Vigorous Physical Activity				
No (ref.)	1.00	REF	1.00	REF
Yes	3.95 ***	(1.94, 8.08)	2.16 ***	(1.47, 3.17)
**Mental Health History/Adverse Childhood Experiences (ACEs)**				
Per each ACE	0.69	(0.48,0.98)	0.79	(0.63, 1.01)
Major Depressive Disorder				
Yes—lifetime (ref.)	1.00	REF	1.00	REF
Never in Life	11.27 ***	(5.22, 24.34)	1.87 *	(1.07, 3.26)
General Anxiety Disorder				
Yes—lifetime (ref.)	1.00	REF	1.00	REF
Never in Life	9.93 ***	(5.02, 19.63)	3.16 ***	(1.92, 5.20)
Drugs and Alcohol Abuse				
Either/Both (ref.)	1.00	REF	1.00	REF
Neither	4.36 ***	(2.11, 9.05)	1.27	(0.82, 1.97)
**Hosmer-Lemeshow**	0.57		0.29	
**Pseudo-R-Square**	13.6		30.7	

*** *p* < 0.001, ** *p* < 0.01, * *p* < 0.05, + *p* < 0.10 but *p* ≥ 0.05; REF = Reference.

## Data Availability

The public use CCHS-MH data is available from Statistics Canada. Please contact your local Statistics Canada research data centre https://crdcn.ca/data/canadian-community-health-survey-mental-health/#tab-page-1 (accessed on 27 November 2022).

## Supplementary Materials

The following supporting information can be downloaded at: https://www.mdpi.com/article/10.3390/ijerph192316337/s1, Table S1: Odds ratio, 95% Confidence Intervals, Unstandardized Coefficient (B), Standard Error of the Mean (S.E.), Wald, and Degrees of Freedom (df) of Absence of Psychiatric Disorders (APD) in past year for those with a History of COPD in a Population-based Sample of Canadians aged 50 and Older (*n* = 703); Table S2: Odds ratio, 95% Confidence Intervals, Unstandardized Coefficient (B), Standard Error of the Mean (S.E.), Wald, and Degrees of Freedom (df) of Complete Mental Health (CMH) in past year for those with a History of COPD in a Population-based Sample of Canadians aged 50 and Older (*n* = 703).
