# Supplementary material for: Flourishing despite Chronic Obstructive Pulmonary Disease (COPD): Findings from a Nationally Representative Survey of Canadians Aged 50 and Older
Source: Int J Environ Res Public Health. 2022 Dec 6;19(23):16337. doi: 10.3390/ijerph192316337 (PMC9735626; doi:10.3390/ijerph192316337)
Supplement: Supplementary file 1 [file ijerph-19-16337-s001.zip › ijerph-1972296-supplementary.pdf]

Supplementary Materials

Table S1. Odds ratio, 95% Confidence Intervals, unstandardized coefficient (B), Standard Error of the Mean (S.E.), Wald, and Degrees of Freedom (df) of Absence of Psychiatric Disorders (APD) in Past Year for Those with a History of COPD in a Population-based Sample of Canadians aged 50 and Older (n = 703).

	Odds Ratio for (APD) "No Past-Year Mental Illness, Substance Dependence or Suicidality"	95% CI	B	S.E.	Wald	df
**Demographics**						
Gender						
Male (ref.)	1	REF				
Female	1.61	(0.71, 3.62)	0.47	0.41	1.31	1.00
Ethnicity						
Visible Minority (ref.)	1	REF				
White	1.51	(0.45, 5.07)	0.41	0.62	0.45	1.00
Age in Decades	1.14	(0.80, 1.62)	0.13	0.18	0.52	1.00
**Socioeconomic Status**						
Education						
No post-secondary degree (ref.)	1	REF				
Has post-secondary degree	0.82	(0.44, 1.55)	−0.20	0.32	0.37	1.00
Household Income						
Lowest 10% of household income (ref.)	1	REF				
11-50%	1.15	(0.46, 2.86)	0.14	0.46	0.09	1.00
Top 50% of household income	1.66	(0.64, 4.28)	0.51	0.48	1.10	1.00
**Social Support**						
Marital Status						
Single/Divorced/widowed (ref.)	1	REF				
Married/Common in Law	2.14 *	(1.11, 4.15)	0.76	0.34	5.12	1.00
Presence of a Confidant						
Strongly Disagree/Disagree (ref.)	1	REF				
Strongly Agree/Agree	7.97 ***	(3.36, 18.93)	2.08	0.44	22.15	1.00
**Physical Health**						
Smoking						
Ever Smoker (ref.)	1	REF				
Never	0.59	(0.22, 1.59)	−0.53	0.51	1.08	1.00
BMI (self-reported)						
No (ref.)	1	REF				
Yes (Obese)	0.63	(0.31, 1.25)	−0.47	0.35	1.77	1.00
Sleep Problems						
Most or all the time sleep problems (ref.)	1	REF				
Never to some sleep problems	1.38	(0.70, 2.72)	0.32	0.34	0.89	1.00
Pain preventing activities						
Pain prevents few/some/most activities (ref.)	1	REF				
No pain or no activity prevented by pain	1.18	(0.58, 2.41)	0.16	0.36	0.20	1.00
**Coping Strategies**						
Spiritual Values						
Not very/not at all important (ref.)	1	REF				
Very/somewhat important	0.52+	(0.24, 1.12)	−0.66	0.39	2.80	1.00
Moderate or Vigorous Physical Activity						
No (ref.) 1.00		REF				
Yes	3.95 ***	(1.94, 8.08)	1.37	0.36	14.22	1.00
**Mental Health History/Adverse Childhood Experiences (ACEs)**	0.69	(0.48,0.98)	−0.38	0.18	4.25	1.00

Per each ACES						
Major Depressive Disorder						
Yes - lifetime (ref.)	1	REF				
Never in Life	11.27 ***	(5.22, 24.34)	2.42	0.39	37.98	1.00
General Anxiety Disorder						
Yes – lifetime (ref.)	1	REF				
Never in Life	9.93 ***	(5.02, 19.63)	2.30	0.35	43.60	1.00
Drugs and Alcohol Abuse						
Either/Both (ref.)	1	REF				
Neither	4.36 ***	(2.11, 9.05)	1.47	0.37	15.68	1.00

*** $p < 0.001$, ** $p < 0.01$, * $p < 0.05$, + $p < 0.10$ but $p \geq 0.05$; REF = Reference.

Table S2. Odds ratio, 95% Confidence Intervals, Unstandardized Coefficient (B), Standard Error of the Mean (S.E.), Wald, and Degrees of Freedom (df) of Complete Mental Health (CMH) in Past Year for Those with a History of COPD in a Population-based Sample of Canadians aged 50 and Older (n = 703).

	Odds Ratio of Complete Mental Health	95% CI	B	S.E.	Wald	df
**Demographics**						
Gender						
Male (ref.)	1	REF				
Female	1.14	(0.74, 1.76)	0.13	0.22	0.37	1.00
Ethnicity						
Visible Minority (ref.)	1	REF				
White	2.76 **	(1.44, 5.30)	1.02	0.33	9.36	1.00
Age in Decades	1.18	(0.96, 1.44)	0.16	0.10	2.45	1.00
**Socioeconomic Status**						
Education						
No post-secondary degree (ref.)	1	REF				
Has post-secondary degree	1.18	(0.81, 1.71)	0.16	0.19	0.70	1.00
Household Income						
Lowest 10% of household income (ref.)	1	REF				
11-50%	0.9	(0.51, 1.59)	−0.10	0.29	0.12	1.00
Top 50% of household income	1.22	(0.68, 2.17)	0.20	0.29	0.44	1.00
**Social Support**						
Marital Status						
Single/Divorced/widowed (ref.)	1	REF				
Married/Common in Law	1.64 *	(1.11, 2.42)	0.49	0.20	6.13	1.00
Presence of a Confidant						
Strongly Disagree/Disagree (ref.)	1	REF				
Strongly Agree/Agree	6.93 ***	(3.32, 14.47)	1.94	0.38	26.53	1.00
**Physical Health**						
Smoking						
Ever Smoker (ref.)	1	REF				
Never	1.21	(0.68, 2.15)	0.19	0.29	0.41	1.00
BMI (self-reported)						
No (ref.)	1	REF				
Yes (Obese)	1.24	(0.81, 1.88)	0.21	0.21	0.99	1.00
Sleep Problems						
Most or all the time sleep problems (ref.)	1	REF				
Never to some sleep problems	1.23	(0.81, 1.87)	0.21	0.21	0.94	1.00
Pain preventing activities						
Pain prevents few/some/most activities (ref.)	1	REF				
No pain or no activity prevented by pain	1.44+	(0.97, 2.12)	0.36	0.20	3.31	1.00
**Coping Strategies**						
Spiritual Values						
Not very/not at all important (ref.)	1	REF				

Very/somewhat important	1.50+	(0.98, 2.29)	0.40	0.22	3.45	1.00
Moderate or Vigorous Physical Activity						
No (ref.) 1.00	1	REF				
Yes	2.16 ***	(1.47, 3.17)	0.77	0.20	15.26	1.00
**Mental Health History/Adverse Childhood Experiences (ACEs)**						
		(0.63, 1.01)	−0.23	0.12	3.59	1.00
Per each ACES						
Major Depressive Disorder	0.79					
Yes - lifetime (ref.)	1	REF				
Never in Life	1.87 *	(1.07, 3.26)	0.63	0.28	4.89	1.00
General Anxiety Disorder						
Yes – lifetime (ref.)	1	REF				
Never in Life	3.16 ***	(1.92, 5.20)	1.15	0.26	20.31	1.00
Drugs and Alcohol Abuse						
Either/Both (ref.)	1	REF				
Neither	1.27	(0.82, 1.97)	0.24	0.22	1.14	1.00

*** $p < 0.001$, ** $p < 0.01$, * $p < 0.05$, + $p < 0.10$ but $p \geq 0.05$; REF = Reference.
